# Wilms' tumour 1‐associating protein inhibits endothelial cell angiogenesis by m6A‐dependent epigenetic silencing of desmoplakin in brain arteriovenous malformation

**DOI:** 10.1111/jcmm.15101

**Published:** 2020-04-13

**Authors:** Lin‐jian Wang, Yimeng Xue, Hao Li, Ran Huo, Zihan Yan, Jie Wang, Hongyuan Xu, Jia Wang, Yong Cao, Ji‐zong Zhao

**Affiliations:** ^1^ Savaid Medical School University of Chinese Academy of Sciences Beijing China; ^2^ China National Clinical Research Center for Neurological Diseases Beijing China; ^3^ Department of Neurosurgery Beijing Tiantan Hospital Capital Medical University Beijing China; ^4^ Center of Stroke Beijing Institute for Brain Disorders Beijing China; ^5^ Beijing Key Laboratory of Translational Medicine for Cerebrovascular Disease Beijing China

**Keywords:** angiogenesis, brain arteriovenous malformation, desmoplakin, m6A, Wnt pathway, Wilms' tumour 1‐associating protein

## Abstract

Brain arteriovenous malformations (AVMs) are congenital vascular abnormality in which arteries and veins connect directly without an intervening capillary bed. So far, the pathogenesis of brain AVMs remains unclear. Here, we found that Wilms' tumour 1‐associating protein (WTAP), which has been identified as a key subunit of the m6A methyltransferase complex, was down‐regulated in brain AVM lesions. Furthermore, the lack of WTAP could inhibit endothelial cell angiogenesis in vitro. In order to screen for downstream targets of WTAP, we performed RNA transcriptome sequencing (RNA‐seq) and Methylated RNA Immunoprecipitation Sequencing technology (MeRIP‐seq) using WTAP‐deficient and control endothelial cells. Finally, we determined that WTAP regulated Desmoplakin (DSP) expression through m6A modification, thereby affecting angiogenesis of endothelial cells. In addition, an increase in Wilms' tumour 1 (WT1) activity caused by WTAP deficiency resulted in substantial degradation of β‐catenin, which might also inhibit angiogenesis of endothelial cells. Collectively, our findings revealed the critical function of WTAP in angiogenesis and laid a solid foundation for the elucidation of the pathogenesis of brain AVMs.

## 1 INTRODUCTION

Brain arteriovenous malformations (AVMs) are characterized by direct anastomosis between the arterial and venous channels without any intervention of the capillaries.^1^ The estimated crude annual detection rate has been reported at 1.3 per 100 000 patient‐years.^2^, ^3^ Along with the advancement of diagnostic techniques, the detection rate of brain AVMs is increasing and add up to an estimated prevalence of approximately 50 cases per 100 000.^4^ Clinically, cerebral haemorrhage, partial or systemic epileptic seizure, and transient ischaemic attack are the most common relevant symptoms,^5^ seriously endangering human health and life. But until now, the pathogenesis of brain AVMs is still unclear.

N6‐methyladenosine (m6A) is identified to be the most common and abundant RNA molecular modification in eukaryotes^6^ and is involved in a variety of metabolic processes of RNA, such as RNA transcription, shearing, nuclear transport and translation ability.^7^, ^8^, ^9^, ^10^, ^11^, ^12^, ^13^, ^14^, ^15^ The methylation modification of m6A is reversible by the implication of the methyltransferases, demethylases and methylated reading proteins. It is well clarified that Wilms' tumour 1‐associating protein (WTAP), acting as the most important regulatory subunit, forms the core methyltransferase complex with METTL3 and METTL14 to catalyse the m6A modification.^16^, ^17^, ^18^, ^19^, ^20^ In mammals, m6A can be reverted to adenosine by the m6A RNA demethylase, FTO and ALKBH5.^21^, ^22^ Furthermore, m6A modification requires the specific RNA binding proteins, also known as ‘readers’, to perform specific biological functions. RNA pull‐down experiments have identified a variety of reading proteins, including YT521‐B homology (YTH) domain‐containing protein, heterogeneous nuclear ribonucleoprotein (hnRNP), IGF2BP proteins and eukaryotic initiation factor (eIF).^23^, ^24^, ^25^, ^26^, ^27^ Recently, an increasing number of studies have shown that m6A modification widely participates in the regulation of multiple biological processes^8^, ^9^, ^28^, ^29^, ^30^, ^31^, ^32^ and exhibited a correlation between aberrant cellular m6A level and diseases.^33^, ^34^, ^35^, ^36^ In addition, previous studies have reported that zebrafish embryos with deficiency of WTAP displayed multiple developmental defects.^19^ However, it is unclear whether WTAP participates in the genesis and progression of brain AVMs.

Desmoplakin (DSP) is a critical component of desmosome and plays a central role in maintaining the structure and stability of the desmosome.^37^ In addition, previous studies have demonstrated that DSP is essential for the mechanical integrity of epithelium and myocardium.^38^, ^39^, ^40^ DSP gene mutations are associated with cutaneous or cardiac defects, such as palmoplantar keratoderma, skin fragility‐woolly hair syndrome, lethal acantholytic epidermolysis bullosa, arrhythmogenic right ventricular cardiomyopathy and Carvajal syndrome.^41^, ^42^ In addition to cell adhesion, DSP has recently been found to be involved in other cellular processes such as proliferation, differentiation and carcinogenesis.^40^, ^43^ Surprisingly, DSP is not only related to epidermal integrity and cardiac function, but also to vascular development. For instance, ablation of DSP results in leaky and/or poorly formed capillaries, limiting embryonic development.^44^, ^45^ Therefore, it is of great significance to explore the relationship between DSP and the pathogenesis of brain AVM.

In the current study, we found that WTAP was down‐regulated in brain AVM lesions compared with normal cerebral vessels, and knockdown of WTAP significantly inhibited tube formation of the human endothelial cells. To thoroughly investigate the specific molecular mechanism, we performed RNA transcriptome sequencing (RNA‐seq) and Methylated RNA Immunoprecipitation Sequencing technology (MeRIP‐seq) to screen downstream targets of WTAP. Finally, we determined that DSP was stabilized via WTAP‐m6A‐IGF2BPs‐dependent manner and participated in the regulation of angiogenesis. In addition, Wnt pathway was repressed due to elevated levels of free Wilms' tumour 1 (WT1) in WTAP‐deficient endothelial cells, which might be involved in the formation of brain AVMs. Our findings determine the mechanism by which WTAP inhibits angiogenesis and provide potential therapeutic targets to prevent the formation or progression of brain AVMs.

## 2 MATERIALS AND METHODS

### 2.1 Patients and samples

Detailed information on patient recruitment and sample preparation could be found in our previous study.^46^ Briefly, from September 2016 to November 2017, we recruited 66 patients with brain AVM and seven patients with epilepsy as a control at Beijing Tiantan Hospital affiliated to Capital Medical University. Brain AVMs samples were collected from consecutive patients undergoing surgical treatment. The clinical diagnoses of brain AVM were confirmed by digital subtraction angiography and histologic evaluation in the hospital's pathology department. In addition, consistent with previous study,^47^ intracranial vascular tissue samples without the typical characteristics of brain AVM had been obtained from 7 patients undergoing temporal lobe resection for epilepsy. Informed consents were obtained from all patients, and this study was approved by the institutional review board of Beijing Tiantan Hospital, Capital Medical University.

### 2.2 Cell culture

The human umbilical vein endothelial cells (HUVECs) were purchased from ScienCell (Carlsbad, CA) and maintained in endothelial cell medium (ECM, ScienCell) supplemented with 5% foetal bovine serum (FBS, Gibco), 100 U/mL penicillin and 100 μg/mL streptomycin.

### 2.3 Gene silencing and expression

The siRNAs targeting specific genes were designed and synthesized by Guangzhou RiboBio (Table S1). Endothelial cells were transfected with siRNAs using Lipofectamine RNAiMAX (Invitrogen). The plasmid expressing Flag‐tagged *Homo sapiens* WTAP was synthesized by Shanghai Genechem Co., Ltd. Constructed plasmid was transfected into the endothelial cells according to the manufacturer's instructions of jetPRIME kit (Polyplus‐transfection). After transfection for 48 hours, endothelial cells were harvested for subsequent mRNA or protein expression analysis.

### 2.4 RNA isolation and qRT‐PCR

Total RNAs were extracted and purified using TRIzol reagent (Invitrogen) according to the manufacturer's instructions. cDNA was reverse transcribed from total RNAs using the PrimeScript™ RT reagent Kit with gDNA Eraser (TaKaRa Co). qRT‐PCR was performed using the SYBR^®^ Premix Ex Taq™ II (TaKaRa) on the QuantStudio™ real‐time PCR system (Applied Biosystems). Primers for specific genes were listed in Table S2. Finally, the relative analysis of gene expression was evaluated using the $2^{-\Delta \Delta C_{T}}$ method.

### 2.5 Western blotting

Endothelial cells were harvested and lysed in RIPA lysis buffer supplemented with protease and phosphatase inhibitors. Protein samples were separated by sodium dodecyl sulphate polyacrylamide gel electrophoresis (SDS‐PAGE) and transferred onto a polyvinylidene difluoride (PVDF) membrane (Merck Millipore). After blocking for 1 hour in 5% skimmed milk, the membranes were incubated with the specific primary antibodies as follows: anti‐WTAP (ab195380; abcam), anti‐β‐Actin (ab8227; abcam), anti‐CTNNB1 (ab32572; abcam), anti‐DSP (25318‐1‐AP; proteintech), anti‐IGF2BP1 (22803‐1‐AP; proteintech), anti‐IGF2BP2 (11601‐1‐AP; proteintech) and anti‐IGF2BP3 (14642‐1‐AP; proteintech). After that, the PVDF membranes were incubated with Horseradish peroxidase‐conjugated anti‐rabbit/mouse IgG (M21002/M21001; Abmart), and then, the immunolabelled proteins were visualized using ECL reagent (Merck Millipore).

### 2.6 Immunofluorescence

The endothelial cells were fixed using 4% paraformaldehyde for 20 minutes. After that, the cells were incubated with 0.3% Triton X‐100 for 10 minutes and blocked non‐specific binding sites with 5% BSA. Next, the cells were incubated with primary antibodies against m6A (202003; Synaptic Systems) or β‐catenin (ab32572, Abcam) overnight at 4°C and subsequently were incubated with Alexa Fluor 594‐ or Alexa Fluor 488‐conjugated goat anti‐rabbit secondary antibody at room temperature for 1 hour. Finally, nuclear staining was performed with DAPI at room temperature, and the cells were observed using EVOS™ FL Auto 2 Imaging System (Invitrogen).

### 2.7 Tube formation assay

Tube formation assays were performed using Ibidi μ‐Slide Angiogenesis (Ibidi) according to the manufacturer's protocol. A total of 15 000 endothelial cells in 50 μL complete media were plated to the inner well of µ‐Slide filled with Matrigel. Then, the μ–Slides were incubated at 37°C as usual. About 24 hours later, the tube formations were imaged under the Fluorescence Inversion Microscope System and analysed using the Image J software.

### 2.8 RNA stability assays

Endothelial cells were transfected with siRNAs against specific genes or negative control siRNA using Lipofectamine RNAiMAX (Invitrogen). Twenty‐four hours after transfection, cells were treated with 10 μg/mL actinomycin D (MCE, HY‐17559) and collected at indicated time points. The total RNAs were extracted by TRIzol (Invitrogen) at indicated time points and analysed by qRT‐PCR. The turnover rate and half‐life of mRNA were estimated according to a previously published paper.^48^

### 2.9 Methylated RNA immunoprecipitation

m6A modifications on specific genes were determined using the Magna MeRIP m6A Kit (Millipore, 17‐10499) according to the manufacturer's instructions. In brief, for MeRIP‐seq, 300 μg total RNAs from control and WTAP‐deficient endothelial cells were chemically fragmented into about 100 nucleotides in length by incubation in fragmentation buffer (10 mmol/L ZnCl2, 10 mmol/L Tris‐HCl, pH 7.0) at 94°C for 3 minutes. The reaction was then stopped with 0.05 mol/L EDTA, followed by magnetic immunoprecipitation with the monoclonal antibody towards m6A. Methylated RNAs were eluted by competition with free m6A and extracted using the RNeasy kit (Qiagen). Thereafter, the library construction and sequencing were performed by Cloud‐Seq Biotech Ltd. Co. Both the m6A‐IP samples and the input samples without immunoprecipitation were used for RNA‐seq library generation with NEBNext^®^ Ultra II Directional RNA Library Prep Kit (New England Biolabs, Inc). The library quality was evaluated with BioAnalyzer 2100 system (Agilent Technologies, Inc). Library sequencing was performed on an illumina Hiseq instrument with 150 bp paired‐end reads. Paired‐end reads were harvested from Illumina HiSeq 4000 sequencer and were quality controlled by Q30. After 3’ adaptor‐trimming and removing low quality reads by cutadapt software (v1.9.3), clean reads of all libraries were aligned to the reference genome (HG19) by Hisat2 software (v2.0.4). Methylated sites on mRNAs (peaks) were identified by MACS software. Differentially methylated sites were identified by diffReps. The raw data have been deposited in GEO database, and the accession number is http://www.ncbi.nlm.nih.gov/geo/query/acc.cgi?acc=GSE142386.

For m6A‐RIP‐qPCR, the total RNAs were fragmented into 300‐nt fragments after incubation in fragmentation buffer at 94°C for 30 seconds and immunoprecipitated by anti‐m6A antibody according to the procedure shown above. One‐tenth of the fragmented RNAs were saved as input control, and the enrichment of m6A was quantified using qRT‐PCR.

### 2.10 Statistical analysis

All experiments were performed at least three independent replicates, and statistical analyses were performed using GraphPad Prism6 software. Statistical significance was calculated by unpaired Student's *t* test. Results are presented as mean ± SEM, and a *P* value of less than .05 was considered statistically significant.

## 3 RESULTS

### 3.1 WTAP is down‐regulated in brain AVMs lesions and is required for angiogenesis

**FIGURE 1:**
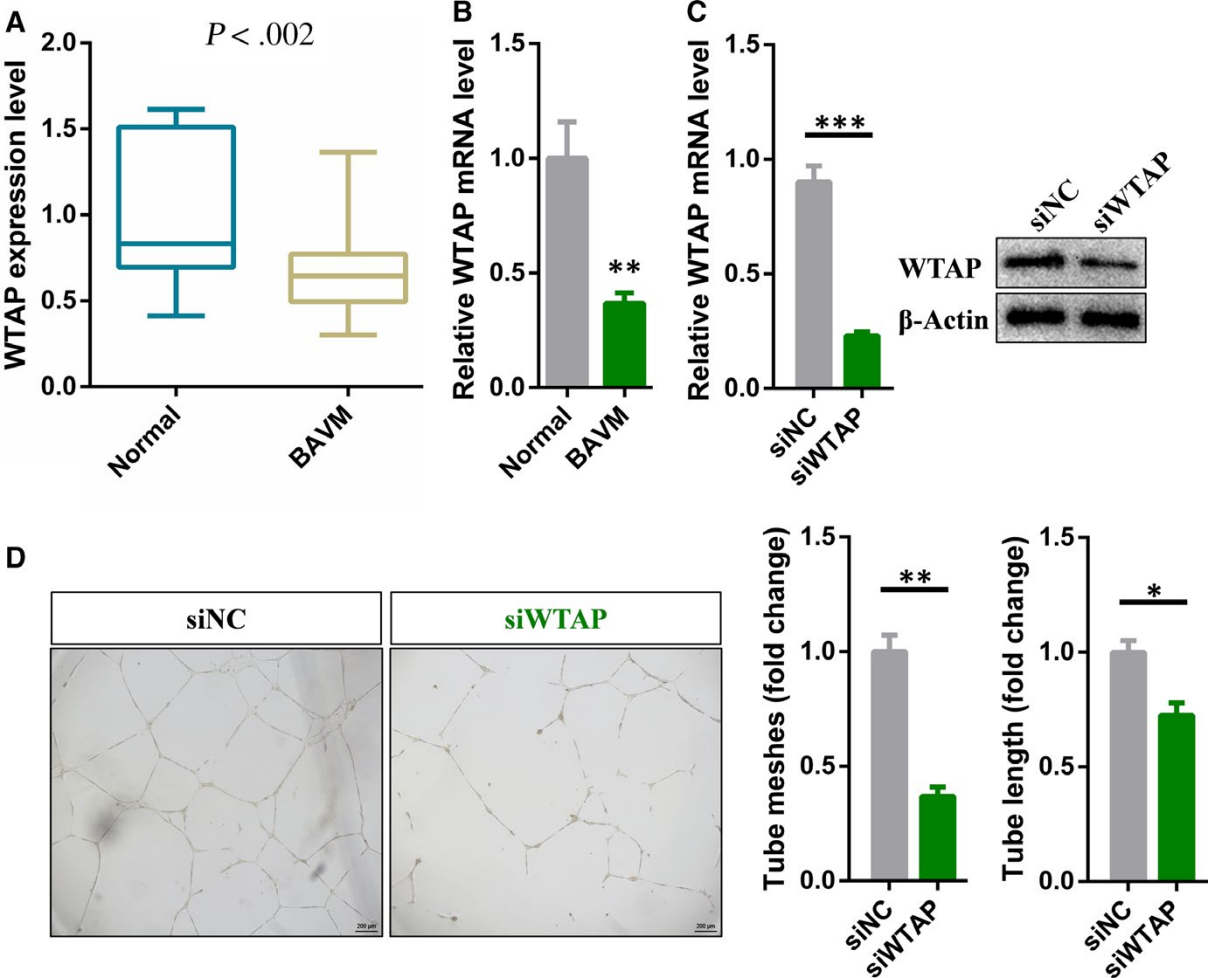
WTAP is down‐regulated in brain AVMs and is required for angiogenesis. A, RNA‐seq showing the expression levels of WTAP in brain AVMs lesions *versus* normal vessels. B, Differential expression of WTAP identified in high‐throughput sequencing was verified by qRT‐PCR. C, qRT‐PCR and Western blot analysis of the knockdown efficiency of WTAP in endothelial cells. D, Representative bright‐field images and statistical analysis of tube formation assay of control and WTAP‐deficient endothelial cells. Data are shown as mean ± SEM of three independent experiments. *P* values were calculated using Student's *t* test. *P < .05; **P < .01; ***P < .001

From September 2016 to November 2017, a total of 66 patients with brain AVM were included in the main study group. Intracranial vascular tissues from seven patients with epilepsy were included as controls. RNA sequencing was performed on collected tissue samples to derive differential gene expression profiles between control and brain AVMs. Through analysing the results of RNA‐seq, we identified a total of 5118 genes that were differentially expressed, and that 2680 and 2438 genes were significantly up‐regulated and down‐regulated in brain AVMs, respectively (Table S3). Surprisingly, we found that although the expression levels of the m6A methyltransferases METTL3 and METTL14 were not significantly different, WTAP was down‐regulated in brain AVMs lesions compared with normal brain vessels (Figure 1A; Figure S1; Table S3). In addition, the expression level of WTAP obtained by high‐throughput sequencing was confirmed by qRT‐PCR (Figure 1B). To investigate the functional changes in vascular endothelial cells that involved in the pathophysiological process of brain AVMs, we knocked down WTAP in human endothelial cells using specific siRNA (Figure 1C). The functional assays showed that knockdown of WTAP significantly inhibited the tube formation of vascular endothelial cells (Figure 1D). Therefore, we concluded that WTAP played a critical role in angiogenesis of vascular endothelial cells.

### 3.2 Analysis of potential targets for WTAP

**FIGURE 2:**
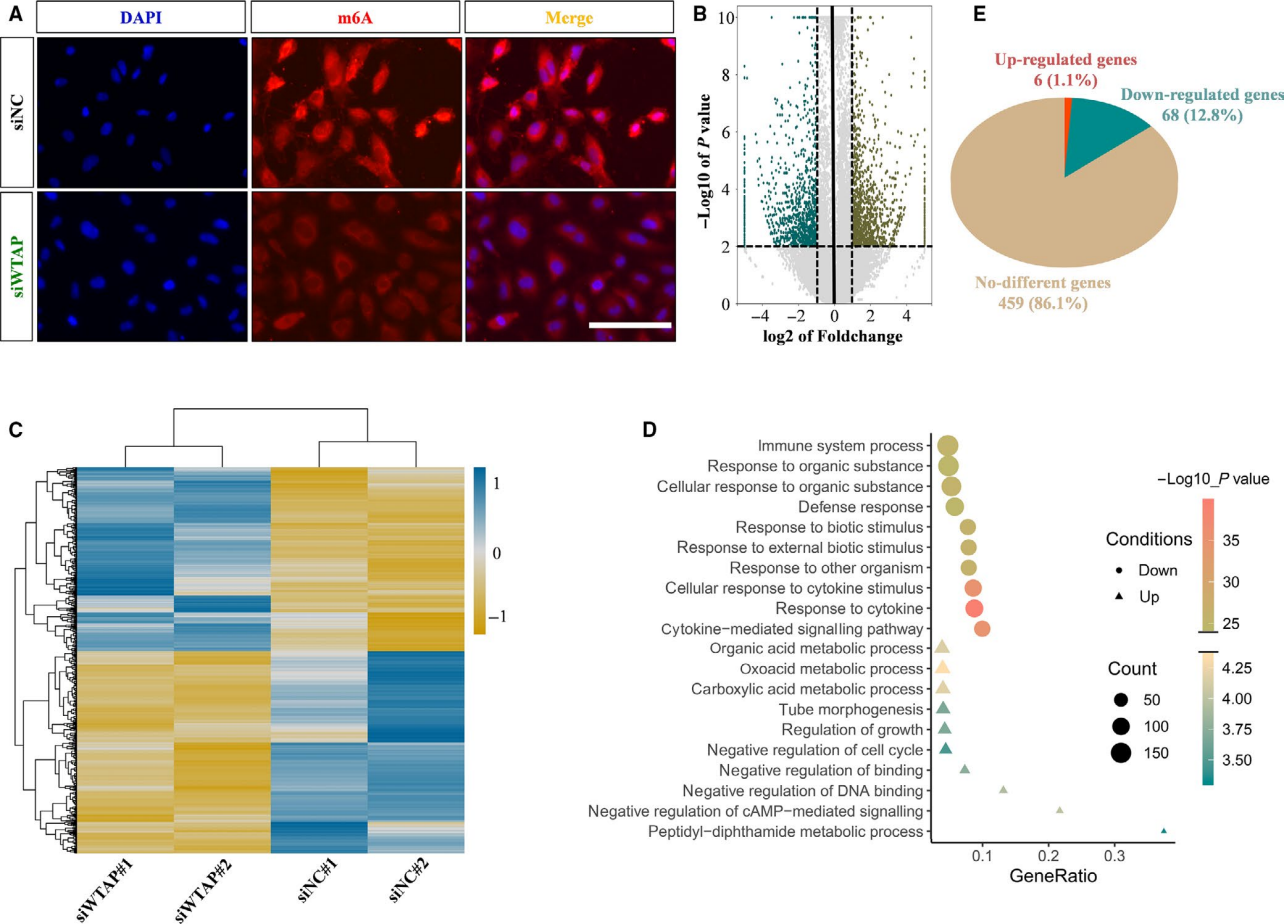
Analysis of potential targets for WTAP. A, Immunofluorescent staining for m6A in control and WTAP‐deficient endothelial cells. B, Volcano map showing the m6A enrichment peaks in WTAP‐deficient endothelial cells compared with control. Significantly increased and decreased peaks (fold change > 2, *P* value < .001) were highlighted in Teal and brown, respectively. C, Heat map depicting differentially expressed genes between control and WTAP‐deficient endothelial cells (fold change > 1.2, *P* value < .05). D, GO analysis of the down‐regulated genes in WTAP‐deficient endothelial cells. E, Pie chart displaying the transcription level of genes with reduced m6A modification

Wilms' tumour 1‐associating protein has been identified to function as a significant regulatory subunit in the m6A methyltransferase complex and plays a critical role in epitranscriptomic regulation of RNA metabolism.^19^ As shown in Figure 2A, silencing of WTAP dramatically reduced the m6A modification level in endothelial cells. To profile the difference in m6A methylation of mRNA, we performed MeRIP‐seq using control and WTAP‐deficient human endothelial cells. The results showed that the level of m6A modification of 533 genes in WTAP‐deficient endothelial cells was significantly lower than that of the control cells (Figure 2B; Table S4). Next, we performed RNA sequencing to fully elucidate the molecular mechanisms of m6A function. About 452 up‐regulated and 495 down‐regulated genes were identified in WTAP‐deficient endothelial cells, respectively (Figure 2C; Table S5). GO enrichment analysis revealed that the up‐regulated genes were enriched in tube morphogenesis, regulation of growth and certain metabolic pathways (Figure 2D). Correspondingly, the down‐regulated genes were enriched in immune‐related pathway (Figure 2D). We then analysed the expression levels of 533 WTAP potential targets in WTAP‐deficient endothelial cells. About 74 targets were shown to be differentially expressed genes, including six up‐regulated and 68 significantly down‐regulated genes (Figure 2E). Therefore, we speculated 74 differentially expressed genes were the directly downstream of WTAP and participated in regulation of tube formation in endothelial cells.

### 3.3 DSP is the downstream target of WTAP

**FIGURE 3:**
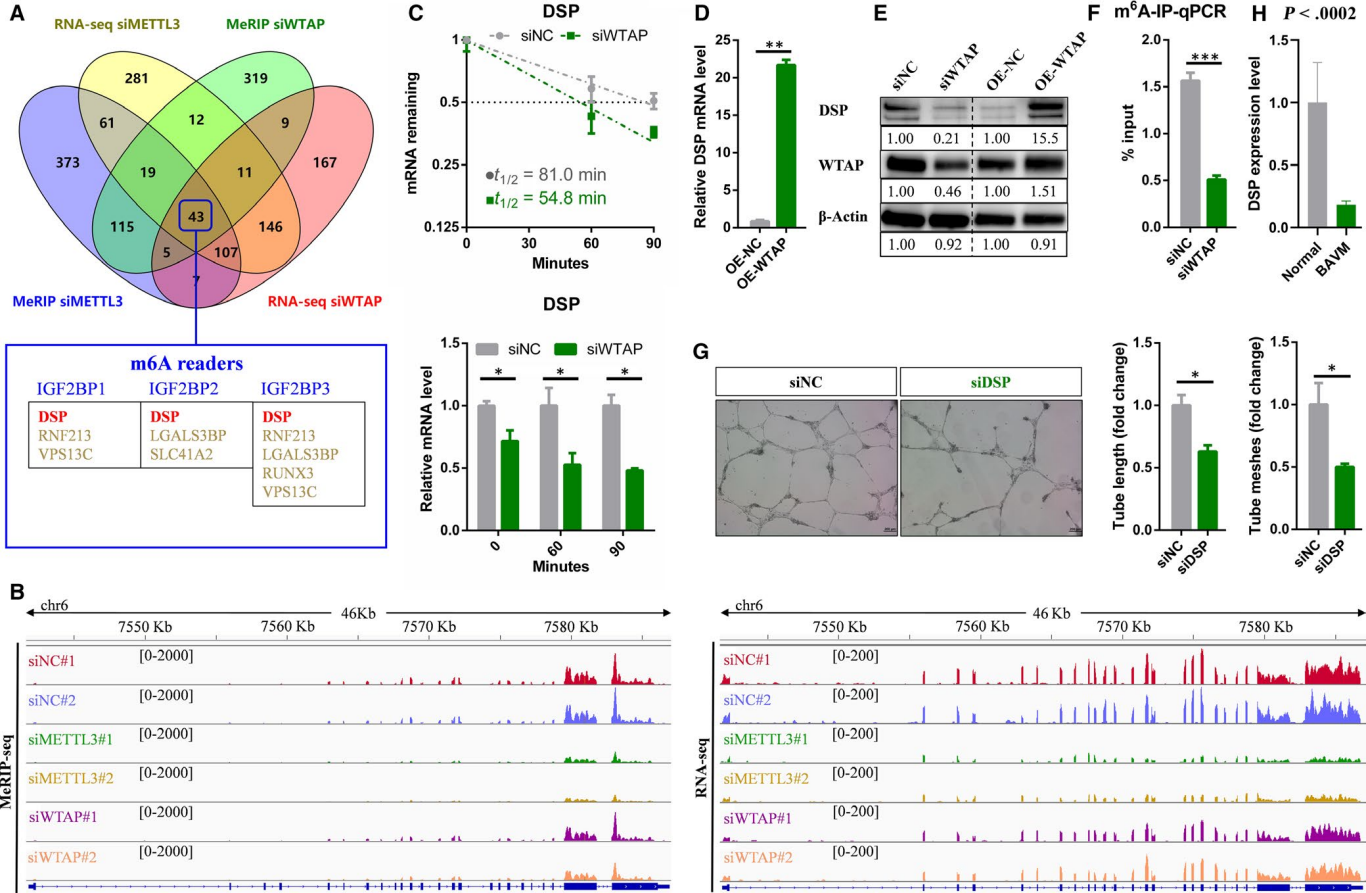
DSP is the downstream target of WTAP. A, Venn diagram showing DSP was the potential target of WTAP. B, Integrative Genomics Viewer (IGV) tracks displaying MeRIP‐seq and RNA‐seq reads distribution in DSP mRNA. C, The half‐life of DSP mRNA in WTAP depletion endothelial cells. D, qRT‐PCR analysis of the mRNA level of DSP in WTAP overexpressing endothelial cells. E, Western blot analysis of indicated proteins in WTAP‐deficient and overexpressing endothelial cells. F, m6A‐RIP‐qPCR displaying m6A enrichment in DSP mRNA in control and WTAP‐deficient endothelial cells. G, Effects of DSP on tube formation of endothelial cells. H, RNA‐seq showing the expression levels of DSP in brain AVMs lesions *vs* normal vessels. Data are shown as mean ± SEM of three independent experiments. *P* values were calculated using Student's *t* test. *P < .05**, *P* < .01; ***P < .001

Previous studies have shown that METTL3 is the most important m6A methyltransferase. Therefore, we also performed MeRIP‐seq and RNA‐seq on METTLE3 knockdown endothelial cells, and the identified differential expressed genes and differentially methylated peaks had been listed in Tables S6 and S7, respectively. In addition, IGF2BPs have been identified as the m6A readers and promote the stability of their target mRNAs in an m6A‐dependent manner.^26^, ^27^ In WTAP‐deficient endothelial cells, most of the differentially expressed genes in 533 transcripts with lower m6A modification levels were down‐regulated. Therefore, we speculated that IGF2BPs as the main m6A readers are involved in the regulation of mRNA stability by WTAP in endothelial cells. Taking these into consideration, we combined the previously published data of IGF2BPs targets identified by RIP and PAR‐CLIP with our MeRIP‐seq and RNA‐seq data on METTL3‐ and WTAP‐deficient endothelial cells for conjoint analysis to further determine the certain target.^26^ Since DSP was also the common target for METTL3 and IGF2BPs, it was selected for further analysis among all the potential WTAP targets (Figure 3A). MeRIP‐seq and RNA‐seq data showed that knockdown of WTAP or METTL3 sharply reduced enrichment of m6A peaks and significantly down‐regulated DSP mRNA levels (Figure 3B; Table S8). Moreover, the results of qRT‐PCR and Western blot confirmed the results of high‐throughput sequencing‐knockdown of WTAP significantly reduced the mRNA level of DSP, while overexpression of WTAP increased the expression level of DSP (Figure 3C‐E). Consistent with MeRIP‐seq results, m6A‐IP‐qPCR results showed that the m6A enrichment in DSP was nearly abolished in WTAP‐deficient endothelial cells (Figure 3F). Furthermore, compared with the control, the half‐lives of DSP mRNA were dramatically shortened, indicating that WTAP can affect the stability of DSP mRNA (Figure 3C). It is well known that m6A peaks in mammalian cells are significantly enriched in RRACH motif (R = G or A; H = A, C or U).^23^, ^49^ By using the online bioinformatics tool m6Avar (http://m6avar.renlab.org/) for analysis, we found that with the exception of the peak distributed in chr6 7756581‐7576689 (HG19), all other identified m6A peaks contained at least one RRACH motif. Collectively, DSP was regulated by WTAP via m6A‐dependent manner. Similar to knockdown of WTAP, the ability of tube formation was drastically decreased in the endothelial cells with silencing of DSP (Figure 3G). Surprisingly, we found DSP was significantly down‐regulated in brain AVMs lesions compared with normal brain vessels (Figure 3H; Table S3). Thus, down‐regulated WTAP repressed the expression level of DSP and ultimately inhibited the tube formation of endothelial cells, which may be related to the pathophysiology of brain AVMs.

### 3.4 The stability of DSP mRNA depends on the m6A reader IGF2BPs

**FIGURE 4:**
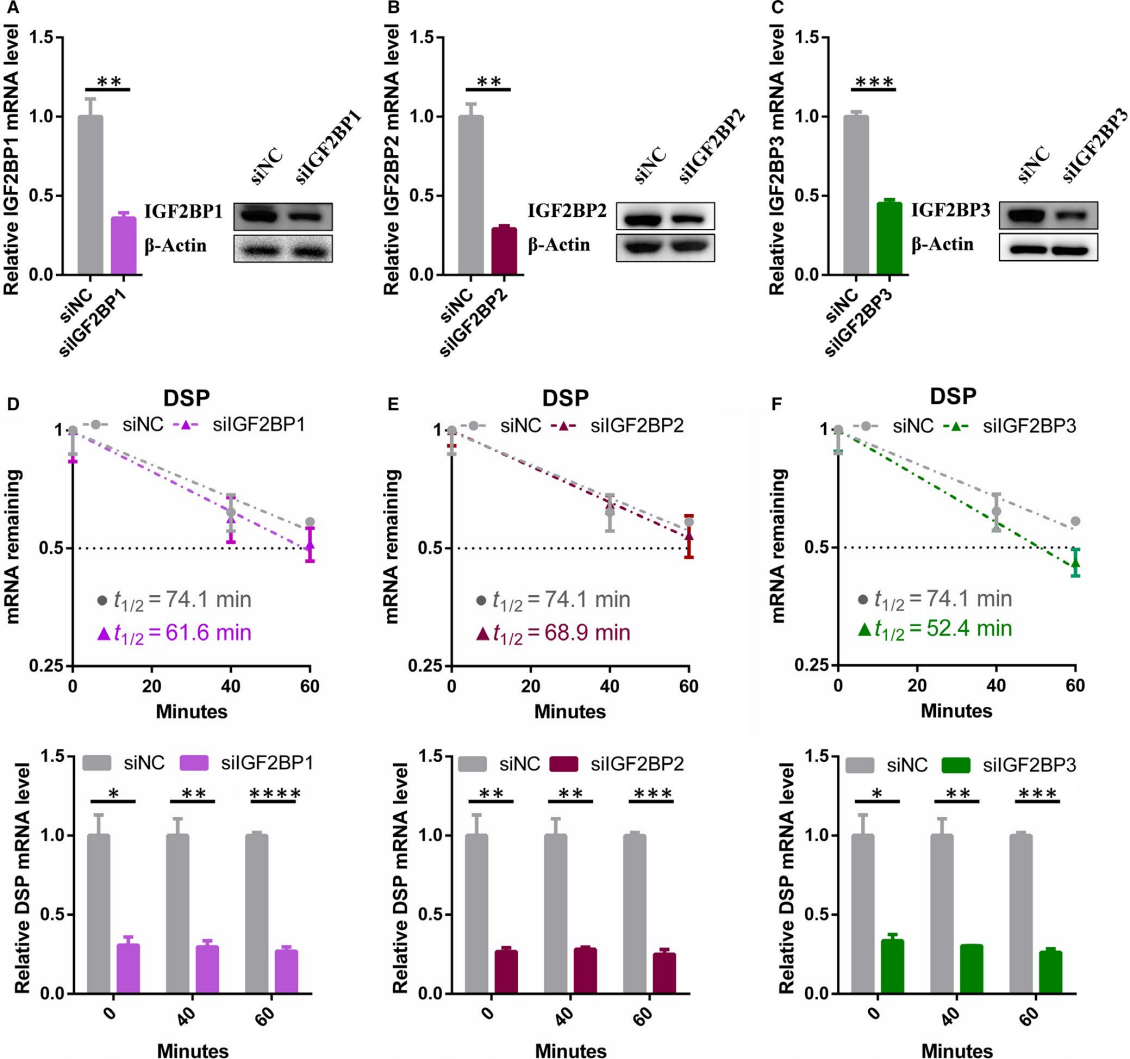
The stability of DSP mRNA depends on the m6A reader IGF2BPs. A‐C, qRT‐PCR and Western blot analysis of the knockdown efficiency of IGF2BP1‐2‐3 in endothelial cells. D, E, The mRNA half‐life of DSP transcript in IGF2BP1, IGF2BP2 and IGF2BP3 depletion endothelial cells (upper); DSP mRNA levels of the knockdown IGF2BPs and control endothelial cells at different time points (lower). Data are shown as mean ± SEM of three independent experiments. *P* values were calculated using Student's *t* test. *P < .05; **P < .01; ***P < .001

Previous study has demonstrated IGF2BP1/2/3 recognize the consensus GGAC sequence through the K homology domains.^26^ We also found that the peak distributed in chr6 7579507‐7581802 (HG19) contained the GGAC motif. Subsequently, we knocked down IGF2BP1, IGF2BP2 and IGF2BP3, respectively, to elucidate whether m6A readers IGF2BPs were involved in the regulation of DSP expression. The knockdown efficiency was identified by qRT‐PCR and Western blot (Figure 4A‐C) Consistent with our hypothesis, DSP mRNA expression levels were significantly reduced after siRNA inhibited any member of IGF2BPs in endothelial cells (Figure 4D‐F). After treatment with actinomycin D, the mRNA levels of DSP were also reduced in IGF2BPs deficient endothelial cells (Figure 4D‐F). However, RNA stability assays showed that the half‐lives of DSP mRNA in IGF2BP1 and IGF2BP3 deficient endothelial cells were dramatically shortened compared with control cells, while the half‐life of DSP mRNA in IGF2BP2 deficient cells was slightly changed (Figure 4D‐F). Taken together, these results suggested that methylated DSP mRNAs could be recognized by the IGF2BP1 and IGF2BP3 to prevent degradation and maintain stability.

### 3.5 WNT signalling pathway is inhibited in WTAP‐deficient endothelial cells

**FIGURE 5:**
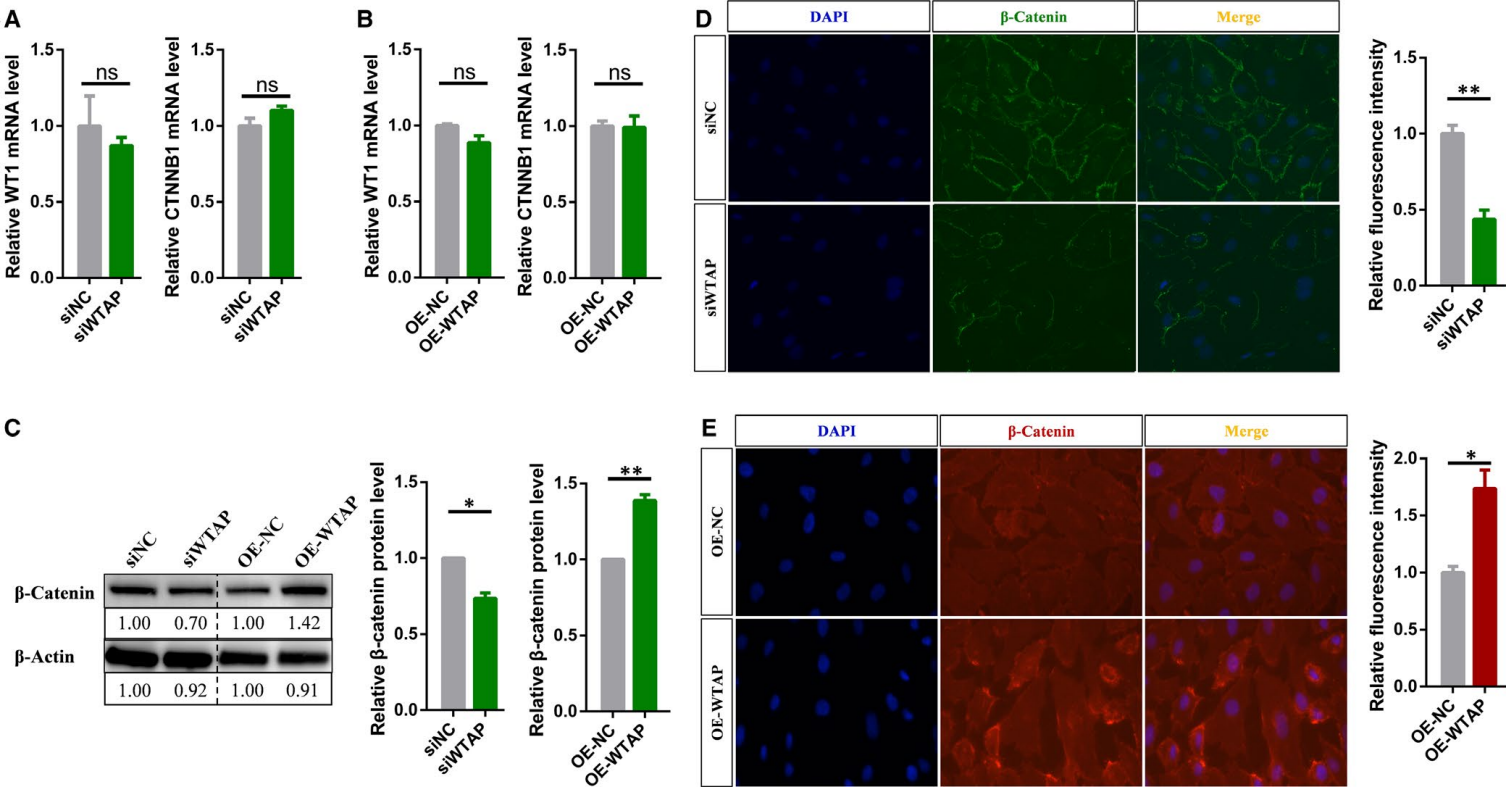
Wnt signalling pathway is inhibited in WTAP‐deficient endothelial cells. A, qRT‐PCR analysis of the mRNA levels of specific genes in WTAP‐deficient or (B) overexpressing endothelial cells. C, Western blot analysis of the protein level of β‐catenin after silencing or overexpressing WTAP. D, Immunofluorescent staining for β‐catenin in WTAP‐deficient or (E) overexpressing endothelial cells. Data are shown as mean ± SEM of three independent experiments. *P* values were calculated using Student's *t* test. *P < .05; **P < .01; ***P < .001

It is well known that Wnt signalling plays a central role in embryonic development, differentiation, cell motility, cell proliferation, adult tissue homeostasis, angiogenesis and so on.^50^, ^51^, ^52^, ^53^ Furthermore, Wnt signalling can activate canonical β‐catenin‐dependent pathway and at least two well‐characterized β‐catenin‐independent pathways, the planar cell polarity (PCP) pathway and the Wnt/Ca2 + pathway.^54^, ^55^ However, Wnt signalling pathway has been reported to be inhibited by targeting the WTAP‐WT1‐TBL1 axis.^56^, ^57^ WT1 is a negative regulator of the Wnt signalling pathway. WTAP can interact with WT1 and form a WTAP/WT1 complex. Therefore, a decrease in WTAP protein levels leads to the release of free WT1, which results in the induction of transducing β‐like protein 1 (TBL1) and ultimately reduces the level of β‐catenin protein.^56^ Therefore, it was worthwhile to explore whether WTAP affects the Wnt signalling pathway through the WT1‐TBL1 axis in endothelial cells. Same as previous research results, silencing or overexpression of WTAP did not affect the expression of WT1 in endothelial cells, but reduced or increased the protein level of β‐catenin, respectively, without affecting its mRNA levels (Figure 5A‐C). Meanwhile, immunofluorescent staining results indicated that knockdown or overexpression of WTAP inhibited or facilitated the translocation of β‐catenin to the nucleus, respectively (Figure 5D,E). In summary, WTAP could modulate the Wnt signalling pathway via releasing free WT1 in endothelial cells.

## 4 DISCUSSION

Brain AVMs, characterized by direct connection between cerebral arteries and veins, have been identified as the important risk factor in fatal symptoms such as cerebral haemorrhage, epilepsy and stroke.^1^, ^5^ But until now, the pathogenesis of brain AVMs is still unclear. In this study, we found WTAP was down‐regulated in the lesions of brain AVMs by transcriptome sequencing (Figure 1A, B). And WTAP could affect the angiogenesis of endothelial cells (Figure 1D). Therefore, down‐regulated WTAP might participated in the pathophysiological process of brain AVMs.

Genetic and epigenetic mechanisms have been elucidated to play an important role in the pathogenesis and development of brain AVMs. For example, somatic activating KRAS mutations were detected in most tissue samples of sporadic non‐familial brain AVM.^47^ In terms of epigenetics, decreased expression of miRNA‐18a in endothelial cells cultured from brain AVMs altered the production of anti‐ and pro‐angiogenic factors.^58^ Furthermore, aberrant epigenetic modifications in the genome of endothelial cells may drive the artery or vein to an aberrant phenotype. Previous studies suggested a significant correlation between DNA methylation levels and brain AVM risk.^59^ Considering that WTAP is an important regulatory subunit of m6A methyltransferase complex,^19^ and can significantly affect the level of m6A modification in endothelial cells (Figure 2A), we speculated that RNA epigenetic modifications‐m6A is also likely to be related to angiogenesis and the formation of brain AVM.

In the current study, we performed MeRIP‐seq and RNA‐seq to reveal underlying molecular mechanisms by which WTAP regulated angiogenesis. Finally, we identified DSP as a downstream target of WTAP‐mediated m6A modification (Figure 3A‐F). DSP is a critical component of desmosome and is essential for maintaining the integrity of tissues, especially those under high mechanical stress, such as epidermis and myocardium.^38^, ^40^ Moreover, previous studies have demonstrated DSP can modulate gene expression, differentiation and microtubule dynamics, indicating that biological functions for this protein exceed its central role in cell‐cell adhesion.^60^, ^61^ Particularly, ablation of DSP leads to leaky and/or poorly formed capillaries, limiting embryonic development.^45^ Here, we confirmed DSP was an essential component of angiogenesis in endothelial cells (Figure 3G), and DSP was significantly down‐regulated in brain AVMs lesions (Figure 3H; Table S3). Consequently, WTAP could regulate DSP expression through m6A modification to affect angiogenesis in brain AVMs. Our findings expanded new developmental processes involving m6A modification in addition to cell differentiation, circadian rhythm, DNA damage response, sex determination, neuronal disorder, infectious diseases and tumorigenesis, and increased our understanding of the pathogenesis of brain AVM. Moreover, there are many other types of modifications in RNA, and their functions are being elucidated. Among them, it is noticeable that m5C, m7G and ac4C have been found to play important regulatory roles in the metabolic process of their modified RNA.^62^, ^63^, ^64^ The role of these epigenetic and other epigenetic factors, such as histone modifications and chromatin states, in the development of AVM requires further research to improve our understanding of disease.

Wilms' tumour 1‐associating protein is a ubiquitously expressed nuclear protein, which was first identified as the partner of WT1, a protein playing an essential role in normal development.^65^ WT1 is a transcription factor that governs the expression of a range of effectors, including genes which regulate the Wnt signalling pathway.^66^ For instance, degradation of WTAP enhances the WT1‐binding activity to induce the expression of TBL1, and this finally results in the degradation of β‐catenin.^56^ In this study, we further confirmed this conclusion: WTAP can antagonize the activity WT1, which negatively regulates the Wnt signalling pathway (Figure 5). The Wnt signalling pathway is one of the pivotal regulatory systems in co‐ordinating endothelial cells behaviour to govern vascular morphogenesis.^67^, ^68^, ^69^ In particular, endothelial‐specific loss of β‐catenin leads to defective vascular remodelling and impairs the development of the embryonic vasculature.^70^ On the basis of the above, we speculated that WTAP could also inhibit angiogenesis by negatively regulating the Wnt pathway.

Overall, our results revealed that WTAP expression level was reduced in lesions of brain AVMs, which would inhibit angiogenesis of endothelial cells. Mechanistically, DSP mRNA was rapidly degraded in WTAP‐deficient endothelial cells due to reduced m6A modification. On the other hand, the lack of WTAP enhanced WT1 activity, thereby repressing the Wnt signalling pathway. These findings will contribute to the elucidation of the pathogenesis of brain AVMs and provide potential targets for treatment.

## CONFLICT OF INTEREST

All authors state that they have no conflicts of interest.

## AUTHOR CONTRIBUTIONS

Lin‐jian Wang, Yong Cao and Ji‐zong Zhao conceived and designed the experiments. Lin‐jian Wang, Ran Huo and Yimeng Xue performed the experiments. Hao Li, Zihan Yan, Hongyuan Xu and Jia Wang contributed data analysis. Lin‐jian Wang wrote the manuscript. Ran Huo, Hao Li, Yimeng Xue, Zihan Yan, Jie Wang, Hongyuan Xu, Jia Wang, Yong Cao and Ji‐zong Zhao revised the manuscript content. Ji‐zong Zhao and Yong Cao approved final version of manuscript.

## Supporting information

Figure S1

Table S1

Table S2

Table S3

Table S4

Table S5

Table S6

Table S7

Table S8

## Data Availability

All authors agreed to share data of this article according to Wiley's Data Sharing Policies.
