# Supplementary figures and images for: Wilms' tumour 1‐associating protein inhibits endothelial cell angiogenesis by m6A‐dependent epigenetic silencing of desmoplakin in brain arteriovenous malformation
Source: J Cell Mol Med. 2020 Apr 13;24(9):4981–91. doi: 10.1111/jcmm.15101 (PMC7205785; doi:10.1111/jcmm.15101)

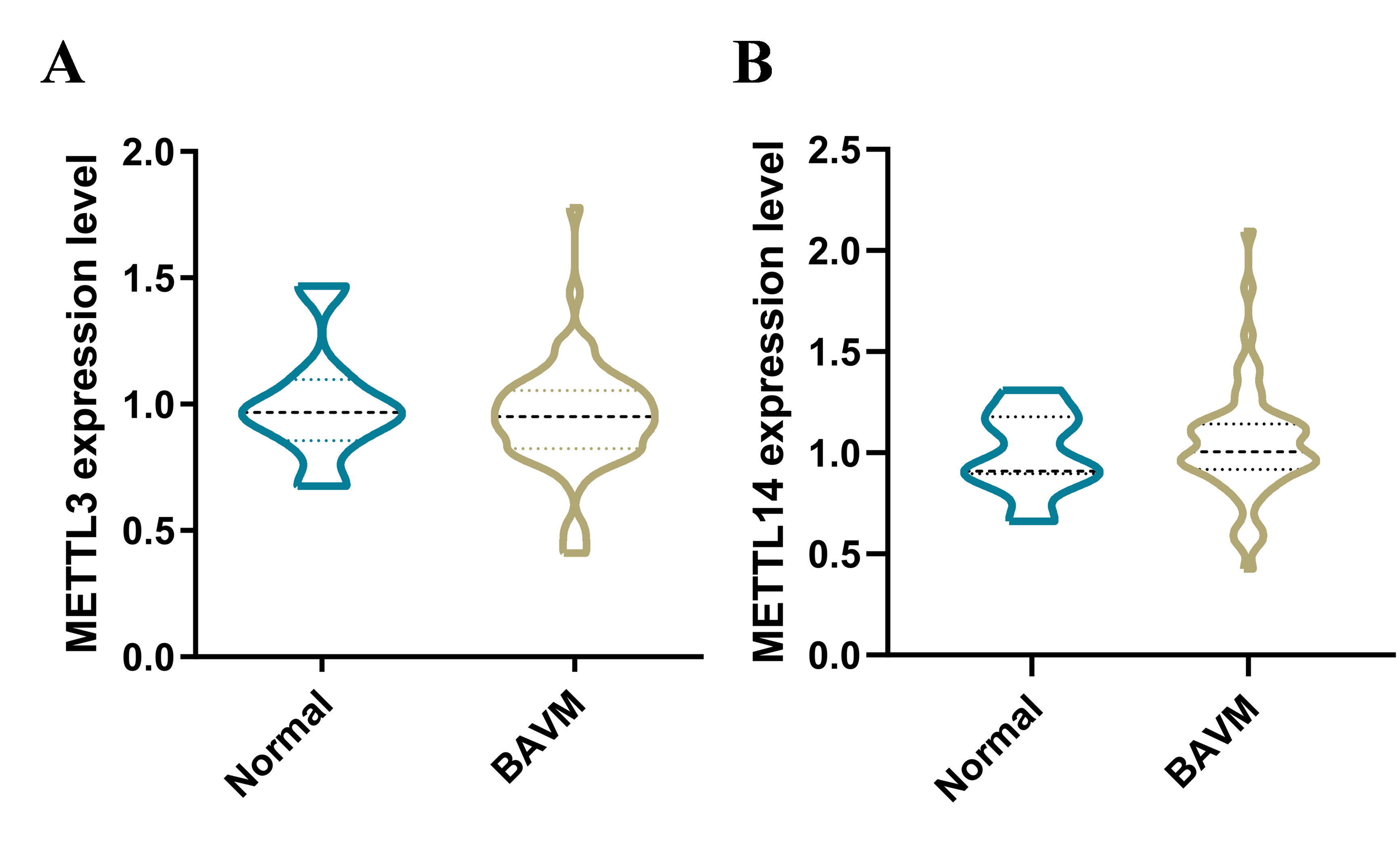

Supplement: Supplementary file 1 — Figure S1 [file JCMM-24-4981-s001.tif]
