# Supplementary material for: Wilms' tumour 1‐associating protein inhibits endothelial cell angiogenesis by m6A‐dependent epigenetic silencing of desmoplakin in brain arteriovenous malformation
Source: J Cell Mol Med. 2020 Apr 13;24(9):4981–91. doi: 10.1111/jcmm.15101 (PMC7205785; doi:10.1111/jcmm.15101)
Supplement: Supplementary file 2 — Table S1 [file JCMM-24-4981-s002.docx]

**Table 1 Primers used for qRT-PCR**

| **Genes** | **Sequences (5'-3')** | |
| --- | --- | --- |
| WTAP | Forward | CTTCCCAAGAAGGTTCGATTGA |
|  | Reverse | TCAGACTCTCTTAGGCCAGTTAC |
| DSP | Forward | ACCAGAACCAGAACACCATC |
|  | Reverse | GGGCAAAACACTCATCCAATTC |
| WT1 | Forward | CCAAATGACATCCCAGCTTG |
|  | Reverse | GTGTGGTTATCGCTCTCGTAC |
| CTNNB1 | Forward | GTTCAGTTGCTTGTTCGTGC |
|  | Reverse | GTTGTGAACATCCCGAGCTAG |
| IGF2BP1 | Forward | AGATGGTGCAGGTGTTTATCC |
|  | Reverse | TTTGGAGTCAGGTGTTTCGG |
| IGF2BP2 | Forward | AATCTCTTCATCCCAACCCAG |
|  | Reverse | ATGACCATCCTTTCGCTGAC |
| IGF2BP3 | Forward | GTTTATCCCAGCTCTATCAGTCG |
|  | Reverse | TCACCATCCTCACTTTAGCATC |
| GAPDH | Forward | AATGACCCCTTCATTGAC |
|  | Reverse | TCCACGACGTACTCAGCGC |
