# Supplementary material for: Wilms' tumour 1‐associating protein inhibits endothelial cell angiogenesis by m6A‐dependent epigenetic silencing of desmoplakin in brain arteriovenous malformation
Source: J Cell Mol Med. 2020 Apr 13;24(9):4981–91. doi: 10.1111/jcmm.15101 (PMC7205785; doi:10.1111/jcmm.15101)
Supplement: Supplementary file 3 — Table S2 [file JCMM-24-4981-s003.docx]

**Table 2 siRNA used in this paper**

| **Targets** | **Sequences (5'-3')** | |
| --- | --- | --- |
| siWTAP | #1 | CACAGAUCUUAACUCUAAU |
|  | #2 | GGGAAAACAUCCUUGUAAU |
|  | #3 | GACCCAGCGAUCAACUUGU |
| siDSP | #1 | GGAGGATAAGCACCGAAGA |
|  | #2 | GCATGATTGACATAGAGAA |
|  | #3 | CGATGACCGTCAGCAGATA |
| siIGF2BP1 | #1 | GGCTCAGTATGGTACAGTA |
|  | #2 | TGAAGATCCTGGCCCATAA |
|  | #3 | GAAGGACGGAACCTGAAGA |
| siIGF2BP2 | #1 | CATGCCGCATGATTCTTGA |
|  | #2 | GAACGAACTGCAGAACTTA |
|  | #3 | AACAGGGACCAAGATAACA |
| siIGF2BP3 | #1 | GCTGAGAAGTCGATTACTA |
|  | #2 | TAAGGAAGCTCAAGATATA |
|  | #3 | TCGGAAACTTCAGATACGA |
| siNC | # | UUCUCCGACGUGUCACGU |
